# Rheological Characterization of Three-Dimensional Neuronal Cultures Embedded in PEGylated Fibrin Hydrogels

**DOI:** 10.3390/gels9080642

**Published:** 2023-08-09

**Authors:** Clara F. López-León, Jordi Soriano, Ramon Planet

**Affiliations:** 1 Departament de Física de la Matèria Condensada, Universitat de Barcelona, E-08028 Barcelona, Spain; clara.fernandez@ub.edu (C.F.L.-L.); jordi.soriano@ub.edu (J.S.); 2 Universitat de Barcelona Institute of Complex Systems (UBICS), E-08028 Barcelona, Spain

**Keywords:** biomaterials, neuronal cultures, rheological characterization, hydrogels

## Abstract

Three-dimensional (3D) neuronal cultures are valuable models for studying brain complexity in vitro, and the choice of the bulk material in which the neurons grow is a crucial factor in establishing successful cultures. Indeed, neuronal development and network functionality are influenced by the mechanical properties of the selected material; in turn, these properties may change due to neuron–matrix interactions that alter the microstructure of the material. To advance our understanding of the interplay between neurons and their environment, here we utilized a PEGylated fibrin hydrogel as a scaffold for mouse primary neuronal cultures and carried out a rheological characterization of the scaffold over a three-week period, both with and without cells. We observed that the hydrogels exhibited an elastic response that could be described in terms of the Young’s modulus *E*. The hydrogels without neurons procured a stable E≃420 Pa, while the neuron-laden hydrogels showed a higher E≃590 Pa during the early stages of development that decreased to E≃340 Pa at maturer stages. Our results suggest that neurons and their processes dynamically modify the hydrogel structure during development, potentially compromising both the stability of the material and the functional traits of the developing neuronal network.

## 1. Introduction

Standard neuronal cultures, in which neurons are seeded and grown on a flat surface, have been extensively used to study neuronal circuits in a controlled environment [1,2,3]. In fields such as physics or medicine, these cultures have helped to understand the complex functional organization of living neuronal networks [4], the emergence of activity patterns and their relation to electrophysiological activity in the brain [5,6], as well as the impact of physicochemical perturbations on neuronal networks’ behavior [7,8].

Despite these efforts, a central debate among the neuroscience community is whether these two-dimensional (2D) neuronal cultures are adequate models to understand the complexity of naturally formed neuronal circuits such as the brain, which combines 2D and 3D organization [9,10]. Indeed, neuronal cultures grown in a 2D environment exhibit a much poorer repertoire of neuronal activity as compared to the brain [11,12,13] due to the fact that cell connectivity is restricted to one plane. Therefore, 3D neuronal cultures have been introduced in the last decade to develop better in vitro models. In typical 3D cultures, cells are embedded in rigid scaffolds, jelly matrices or a combination of both, facilitating brain–like neuronal development [14,15,16,17] and the emergence of organizational features such as modularity [15,18,19]. Although different studies have shown that the 3D environment favors a richer neuronal morphology and network activity as compared to standard 2D preparations [20,21], reproducing the complexity of in vivo cell–cell as well as cell–matrix interactions remains a challenge.

The central problem when preparing 3D neuronal cultures is the selection of the bulk material in which the neurons will grow. This material needs to mimic the brain extracellular matrix (ECM), a fibrous network with a complex distribution of proteoglycans, such as hyaluronic acid and elastic fibers embedded in a highly hydrated environment [22,23]. The brain ECM is so unique that it allows for the efficient transport of molecules, nutrients and metabolic waste while providing structural support to the neurons. Thus, the chosen bulk material should be sufficiently permeable for molecular transport and exhibit adequate mechanical properties to support a stable neuronal network formation [24]. Mechanical properties such as stiffness as well as porosity are traits that may be central to regulate the capacity of the neurons to shape a rich arborization and project axons at long distances.

Among all possible ECM-mimicking materials, *hydrogels* have received special attention due to their highly hydrated structure, which is important since about 73–85% of the human brain mass is water [25]. Hydrogels with a natural origin include collagens [18,26], hyaluronic acid-based hydrogels [27,28,29], chitosan gels [30,31] and fibrin hydrogels [32]. Although all of them have emerged as attractive candidates for neuroscience applications, the semisynthetic PEGylated fibrin hydrogels have particularly caught the attention of researchers in neuroengineering and regenerative medicine [33,34]. The reasons for such an interest include their excellent properties such as their biocompatibility and ease of manipulation [32], and their inherent synthetic nature enhances their resistance to degradation and overall favors long–term stability. PEGylated fibrin hydrogels are also transparent, enabling the study of the dynamic and functional characteristics of embedded neural networks through calcium–imaging techniques. Additionally, these hydrogels hold the potential for patient–specific applications, as fibrinogen and thrombin can be directly sourced from patients, allowing for customization and personalized approaches in medical applications.

Despite these advantages, the physical characteristics of PEGylated fibrin hydrogels are still poorly understood, particularly in the context of developmental neuroscience, where neurons and connections continuously evolve within the hydrogel matrix and therefore may change their structural and mechanical properties. To shed light on this quest, here we introduce a rheological protocol to investigate the appropriateness of PEGylated fibrin hydrogels for 3D neuronal cell culturing. Rheological analyses were performed with and without living neurons to evaluate whether neuronal development was altering the hydrogel structure. In either scenario, the hydrogels’ behavior was monitored for 20 days to evaluate possible degradation over time. This exploration was combined with immunostaining techniques to quantify the neuronal arrangement within the hydrogel structure. The results indicate that the investigated hydrogels exhibit an elastic response that can be quantified through the Young’s modulus *E* and whose evolution suggests the formation of a 3D neuronal structure within the hydrogel that extends and grows throughout the volume of the scaffold. We also observed that the presence of cells in the hydrogel increased its stiffness during the first days in culture and then gradually decreased as the neurons developed, suggesting that the growth of the axons and dendrites irreversibly altered the microscopic structure of the hydrogel.

## 2. Results and Discussion

### 2.1. Characterization of Hydrogels’ Viscoelastic Behavior

**Figure 1:**
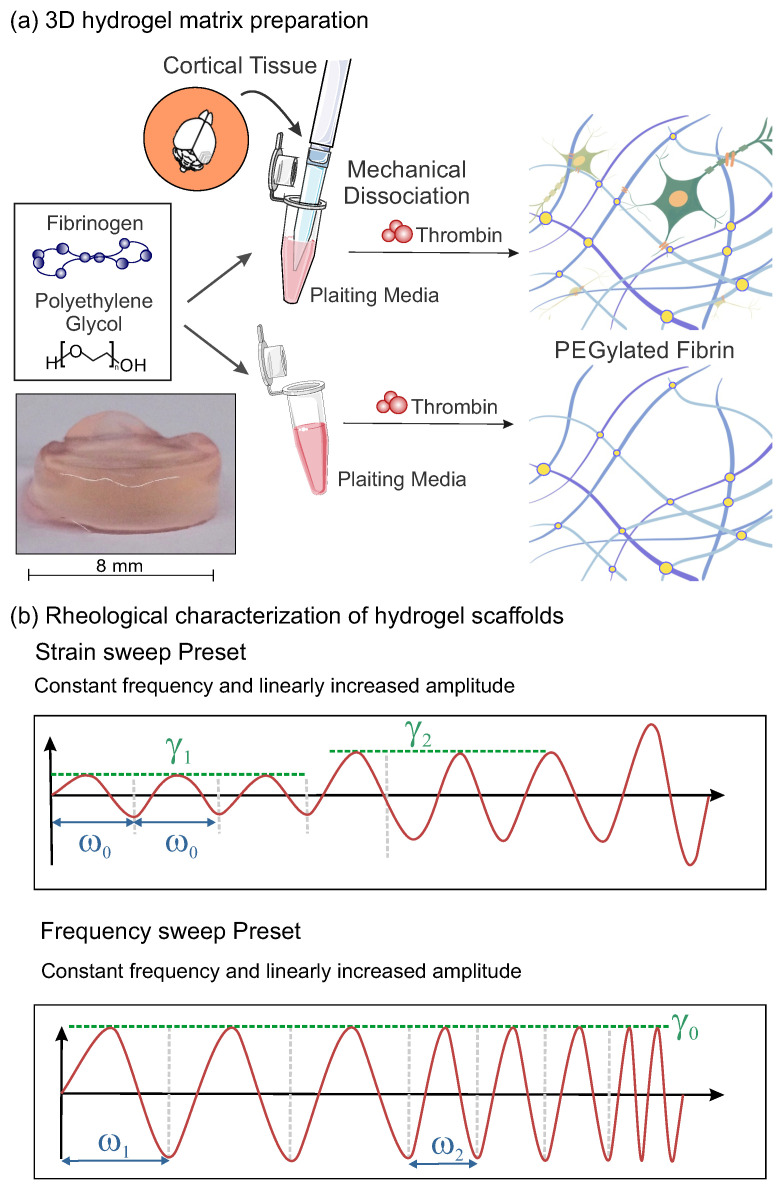
Overview of neuronal cultures preparation and rheological protocols. (**a**) Sketch of the preparation of PEGylated fibrin hydrogel, with and without cells. Final appearance of the PEGylated fibrin hydrogel prepared in Soriano’s Lab (left). (**b**) Established preset protocol for the mechanical characterization of hydrogel samples: strain sweep test with constant $\omega_{0}$ (blue) and varying γ (green) and frequency sweep test with varying ω (blue) and constant $\gamma_{0}$ (green). Red line represents the controlled shear deformation that oscillates sinusoidally.

We considered PEGylated fibrin hydrogels that were studied either as a bulk material or loaded with neurons (Figure 1a). For the latter, a calcium-imaging assay was carried out before any measurement to verify that the embedded neurons were healthy and spontaneously active. We then used rheology to characterize both types of hydrogels since it provides direct information about the bulk mechanical properties [35,36]. The Small Amplitude Oscillatory Shearing (SAOS) rheology (Figure 1b) was used since it applies small deformations, allowing us to characterize the mechanical properties of the hydrogels without damaging or modifying their structure [37]. As described in Methods section, three main rheological tests were carried out, namely *time sweep* to evaluate the hydrogel’s stability during a 15 min measurement at controlled shear deformation, $\gamma \left(t\right)=\gamma_{0}\sin\left(\omega_{0}t\right)$, with $\gamma_{0}$ being the amplitude strain and $\omega_{0}$ the frequency of the oscillation, *strain sweep* to characterize the hydrogel’s response to a varying amplitude strain γ with a fixed frequency $\omega_{0}$ and *frequency sweep* to characterize the hydrogel’s response to a varying frequency ω and fixed amplitude strain $\gamma_{0}$. In order to understand the experimental results, it must be noted that the accuracy of the equipment is an important constraint when designing the measurements since it bounds the range of exploration. For the HR–2 Discovery rheometer, the lower measurable limit corresponds to a 2 nN·m of the oscillation torque. According to this limit, some extracted values of $G^{\prime }$ and $G^{\prime \prime }$ from the strain sweep test were considered unreliable and therefore discarded.

**Figure 2:**
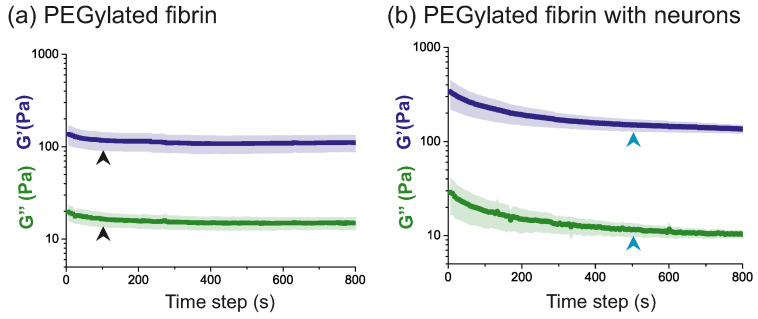
Time sweep results for (**a**) PEGylated fibrin hydrogels and (**b**) PEGylated fibrin hydrogels with neurons. Parameters $\omega_{0}$ and $\gamma_{0}$ were fixed to 2π rad/s and 5%, respectively. Results show that samples reached equilibrium few minutes after the oscillatory effort was applied (arrowheads). Data are the average over 3 sample repetitions, and shadings are the corresponding standard error.

Figure 2 shows the results obtained from the time sweep test for samples measured one day after preparation, i.e., at day in vitro (DIV) 1. The data points were obtained after averaging three different repetitions with the corresponding standard error.

As shown in Figure 2a, the hydrogel samples exhibited a stable response ≃100 s after the deformation was applied (black arrowheads). The time taken to reach stability was about five times larger for the hydrogels with neurons (Figure 2b, blue arrowheads), meaning that the hydrogels with cells took longer to reach an equilibrium state after been subjected to an oscillatory deformation. We conjecture that the larger relaxation time is associated with the interwoven neuronal and hydrogel networks. Additionally, we note that the samples were already cured when performing the test, and therefore no new structural links were formed within the hydrogel that could be ascribed to the longer relaxation time.

The time sweep test results were reproducible across samples, with a consistent $G^{\prime }$ > $G^{\prime \prime }$ along the explored range. We typically measured a $G^{\prime }≃100$ Pa for the PEGylated fibrin scaffolds in the stable regime and $G^{\prime }≃200$ Pa for those samples containing neurons, indicating that the presence of the cells stiffened the scaffold. A $G^{\prime }$ value around 100 Pa was also reported in [38] for fibrin itself, which indicates that our protocol was properly adapted to measure the PEGylated fibrin hydrogel samples and that the results are consistent with the literature.

**Figure 3:**
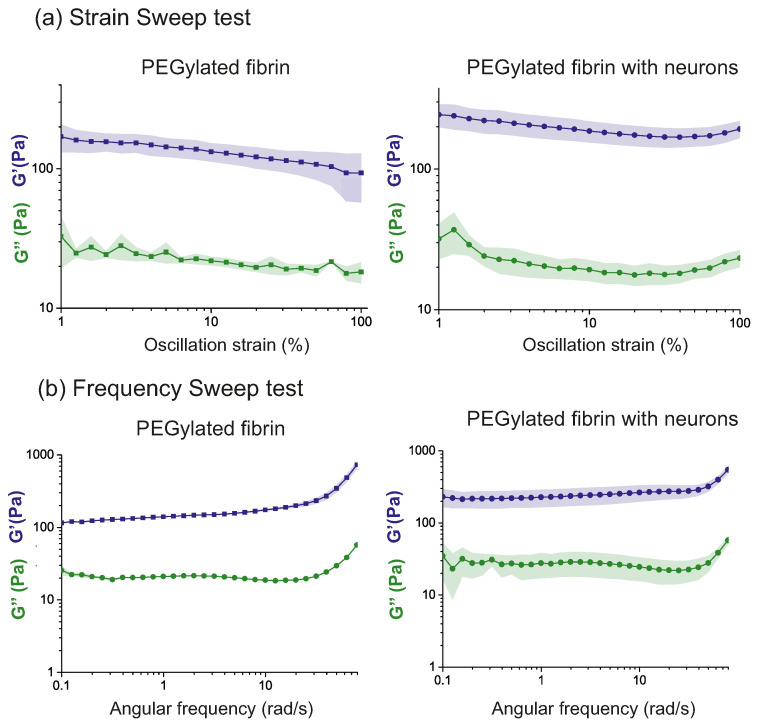
Viscoelastic behavior characterization of hydrogel samples with and without cells at DIV 1. The plots show the storage ($G^{\prime }$) and loss modules ($G^{\prime \prime }$), averaged over 3 repetitions with the corresponding standard errors. (**a**) Strain sweep test for PEGylated fibrin gels (left) and PEGylated fibrin gel with neurons (right), with $\omega_{0}=2\pi$ rad/s and γ∈[1,100]%. (**b**) Frequency sweep test for PEGylated fibrin gels without neurons (left) and with neurons (right), with $\gamma_{0}=5$% and ω∈[0.1,80] rad/s.

The strain sweep test was then performed to identify the linear viscoelastic region for the PEGylated fibrin hydrogels and to study in more detail whether the presence of the cells affected it. As shown in Figure 3a, the two types of samples (with and without cells) exhibited a small negative drift as the strain γ grew. Since the changes in $G^{\prime }$ and $G^{\prime \prime }$ were small enough in the region between the 1 and 10% strain, we considered them to be independent of γ.

We then carried out a frequency sweep test for the samples with and without cells at $\gamma_{0}=5$%, as shown in Figure 3b. This frequency test is key to characterize the hydrogel structure since it informs us about the crosslinking grade of the internal structure of the hydrogel as well as whether the crosslinking reaction is reversible or not. As highlighted before, the absence of a crossing between the $G^{\prime }$ and $G^{\prime \prime }$ curves during the frequency sweep reveals a nonreversible chemically crosslinked structure [36], which comes together with the enzymatic reaction between thrombin and fibrinogen to form the fibrin hydrogel structure. The observed $G^{\prime }$ and $G^{\prime \prime }$ curves in Figure 3b are typical for a hydrogel structure [39], both exhibiting a plateau (despite small drifts or fluctuations) for low oscillation frequencies ω≲10 rad/s that give evidence of a stable, crosslinked network whose values can be related to the stiffness of the hydrogel [36]. The frequency tests also showed that $G^{\prime }>G^{\prime \prime }$ for all frequencies, with $G^{\prime }≃100$ Pa for the PEGylated fibrin and $G^{\prime }≃200$ Pa for the hydrogels seeded with neurons. Additionally, for all the hydrogels investigated, the mean value of δ, as derived from Equation (3) in Methods section, was δ≃0.1 rad for both hydrogel preparations, a value that is consistent with an elastic response.

### 2.2. Time Evolution of PEGylated Fibrin Hydrogels

To use PEGylated fibrin hydrogels as scaffolds for neuronal cultures, it is essential to understand the aging of the gels with time as well as the impact of the neuronal network on their mechanical responses and overall stability. Indeed, knowledge of the stability of the scaffolds with time is crucial to prepare cultures with the appropriate lifespan and helps elucidate the impact of neuronal development and connectivity formation across the whole system. With this aim, we conducted a study on the temporal evolution of the mechanical properties of the hydrogel samples, with and without neurons. Since the values of $G^{\prime }$ exceeded $G^{\prime \prime }$ by a decade (see Figure 3), the elastic response dominates over the viscous behavior. Thus, although both contributions exist, the hydrogels are predominantly elastic. We also note that, due to this elastic dominance, we did not show the plots of $G^{\prime \prime }$ in further analyses.

**Figure 4:**
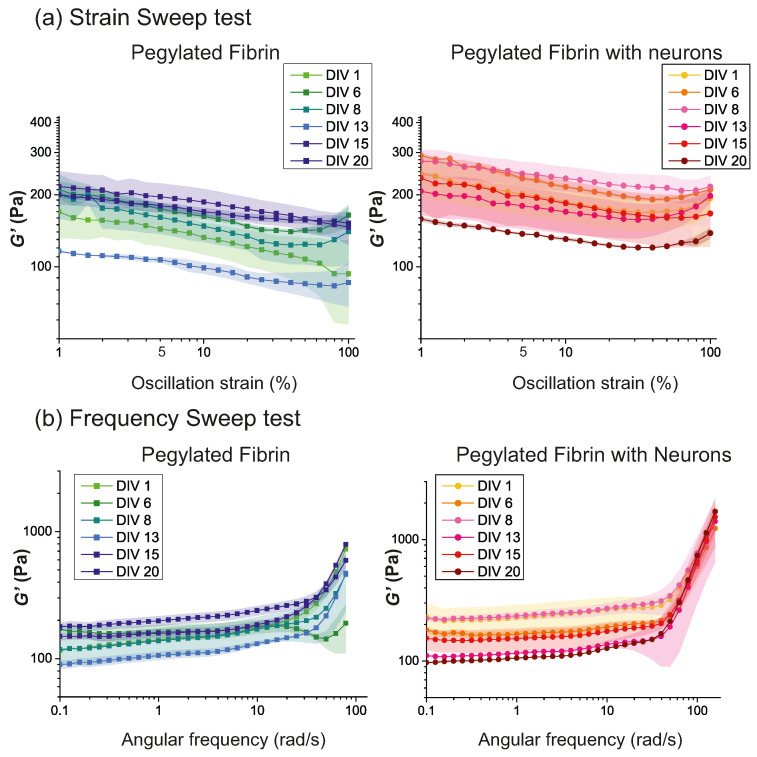
Evolution of PEGylated fibrin hydrogels with and without cells from DIV 1 to DIV 20. (**a**) Strain sweep test, with $\omega_{0}=2\pi$ rad/s and γ∈[1,100]%. (**b**) Frequency sweep test, with $\gamma_{0}=5$% and ω∈[0.1,100] rad/s. In all panels, $G^{\prime }$ values were averaged over 3 repetitions with the corresponding standard error shown as colored shadings. Data corresponding to PEGylated fibrin hydrogels without cells are shown on the left, and data with the inclusion of cells on the right.

Time sweep tests were performed as the first step to verify the structural stability of the samples during the aging process when subjected to an oscillatory force. Next, strain sweep tests were performed on the samples with and without cells from DIV 1 to DIV 20 (Figure 4a). In both cases, the results showed a large sparsity across different DIVs, with the curves crossing one another within a range in $G^{\prime }$ values of 80–220 Pa for the cell-free hydrogels and between 140–300 Pa for the hydrogels with neurons. We note that, for the case of DIV 1, as exemplified in Figure 3a, the $G^{\prime }$ values exhibited as small negative drift with γ. This drift was also observed in the other DIVs along with a similar trend; i.e., the relative vertical position of the curves was maintained along γ. This stability in behavior along the DIVs allowed us to take a fixed value of $\gamma_{0}=5$% for the frequency sweep test and for all the DIVs studied.

With this consideration in mind, Figure 4b shows the results obtained for the frequency sweep tests conducted at $\gamma_{0}$ = 5%. Remarkably, the gel-characteristic low-frequency plateau (with a slight positive drift) was observed in all the samples at different DIVs for frequencies up to ω≃30 rad/s, indicating that neither the presence of cells nor the aging process induced substantial qualitative changes in the elastic behavior of the hydrogel matrix. We note, however, that the drift along ω was more pronounced for the cell–free hydrogels, suggesting that the network matrix stabilized the hydrogel’s response.

We also observed, as shown in Figure 4b, a strong thickening of the hydrogels at large frequencies (ω>50 rad/s) and for all the studied DIVs. This thickening at high–frequency oscillations could be related to the failure of the gel’s network to rearrange when subjected to fast oscillations, i.e., short-time-scale perturbations [40]. We note, however, that high–frequency perturbations may induce inertia effects [41,42] in the response of the hydrogel, and thus the observed thickening could reflect both the network’s difficulty at rearranging itself and the inertial forces from the experimental system.

**Figure 5:**
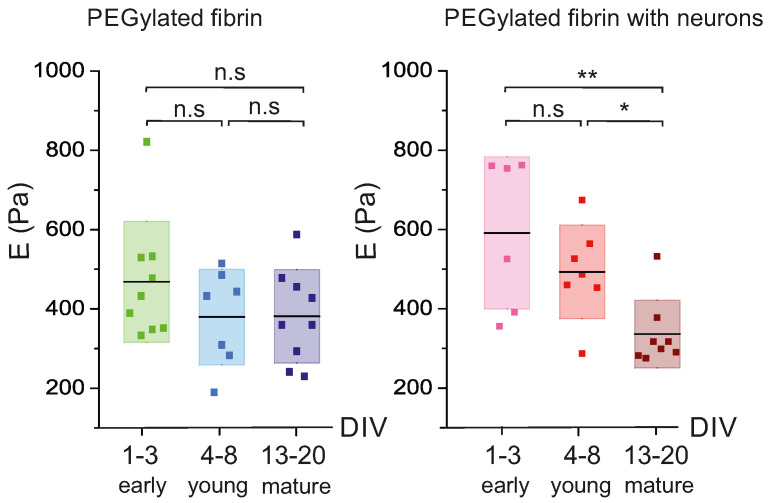
Evolution of the Young’s modulus *E* with time for PEGylated fibrin hydrogels with and without neurons. Data are plotted as boxplot distributions of *E* values in representative stages of evolution, namely early (DIV 1–3), young (DIV 4–8) and mature (DIV 13–20). Each dot in the boxplots is an experimental repetition. The black lines mark the mean of the distribution and the color boxes mark the standard deviation. * *p* < 0.05, ** *p* < 0.01 (Student’s *t*-test).

To gain a deeper understanding of the evolution of the hydrogels’ elasticity, we used the frequency test results ($G^{\prime }$ and $G^{\prime \prime }$) to calculate the Young’s modulus *E* of the PEGylated fibrin hydrogels by using Equations (1) and (2) in the Methods section. The outcomes are shown as boxplots in Figure 5, which display the distribution of *E* values divided into three evolutionary stages (early ‘e’, young ‘y’ and mature ‘m’) for the hydrogels with and without cells. The mean value of the Young’s modulus for each stage is indicated by a black horizontal line.

The distributions shown in the boxplot reveal that the presence of cells exert an effect on the internal structure of the hydrogels, altering the stiffness of the matrix. In the hydrogels without neurons, the Young’s modulus remained approximately constant in terms of the experimental variability during development, with E=420±130 Pa (mean ± standard deviation of the mean) ($p_{e-y}=0.23,\;p_{y-m}=0.97,\;p_{e-m}=0.19$), demonstrating that the PEGylated fibrin hydrogels generate highly stable scaffolds. By contrast, for the hydrogels with neurons, the mean value of *E* within each evolutionary stage gradually decayed, from E=590±210 Pa at the early developmental stages to E=340±100 Pa at the mature ones, a decrease of practically a factor of two that was statistically significant ($p_{e-y}=0.28,\;p_{y-m}=0.011,\;p_{e-m}=0.0055$). This substantial decrease in *E* is puzzling. Intuitively, one would expect that the formation of a neuronal network within the hydrogel strengthens its internal cohesion and makes it less deformable (higher *E*), but we observed the opposite. Thus, we hypothesize that the neurons altered the hydrogel microscopic organization during network formation, either through physical damage from growing axons and dendrites or from enzymatic action from neurons.

### 2.3. Immunostaining Results

**Figure 6:**
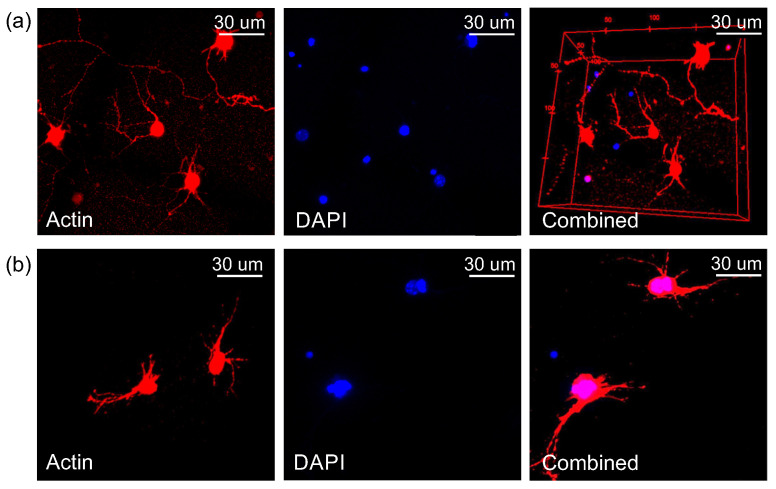
Immunostaining images of neurons within the 3D PEGylated fibrin hydrogel scaffolds showing actin in red (left) and nucleus in blue (center); the panels on the right are the merged images of both channels, showing concurrently the actin filaments and cell nuclei in the 3D culture. In the merged image, pink represents the overlapping of red (actin) and blue (nucleus). (**a**) Sample 1 of PEGylated fibrin hydrogel with neurons at DIV 7, (**b**) Sample 2 of PEGylated fibrin hydrogel with neurons at DIV 7.

The neuronal cultures grown within the PEGylated fibrin hydrogel were immunostained to examine the formation of the neural connections across the volume, providing evidence of healthy neuronal development as well as the neurons’ capacity to extend processes and navigate beyond a single plane within the hydrogel (Figure 6). Actin was marked in red by using Alexa568 conjugated as a secondary antibody, together with a CellMask Red Plasma Membrane Stain that identified every cell body (the cytoskeleton and soma). The cell’s nucleus was marked in blue with DAPI. These markers revealed that the neurons were extensively distributed throughout the entire volume of the hydrogel, forming connections along all three axes.

Due to the difficulty of implementing immunostaining techniques in volumes, it was not possible to obtain results for the evolution of the neuronal network during development. Nevertheless, we concluded that the neurons established connections across different layers of the hydrogel matrix, as shown in Figure 6, which depicts two different samples of the PEGylated fibrin seeded with neurons (a) and (b) at DIV 7. From these images, we measured that the axons had a typical diameter of 1 μm that could easily extend beyond 50 μm. These observations are compatible with the pore size of our hydrogels, in the range 2–8 μm in diameter according to the data provided by Zhang et al. [39], who investigated the structural properties of PEGylated fibrin hydrogels similar to ours by using scanning electron microscopy, SEM. Additionally, neurons’ morphology corresponds to healthy cells, a result that, together with the observation of spontaneous activity through calcium imaging, indicates that the neurons developed healthily and established a well-connected network.

### 2.4. Discussion

Building a 3D microenvironment for cell culturing is crucial to design in vitro models that better emulate the physiology and connectivity of in vivo circuits such as the brain [20]. However, reproducing the complexity of in vivo cell–cell as well as cell–matrix interactions remains a challenge, particularly due to the difficulty of choosing adequate ECM–like materials that support cell development.

To advance in this quest, here we investigated the mechanical properties of hydrogels, a family of synthetic materials widely used in neuroscience given their versatility in tailoring both chemical and mechanical properties to fit particular requirements. We focused our attention on PEGylated fibrin, a semisynthetic hydrogel made of polyethylene glycol (PEG) and a fibrin gel. On the one hand, PEG is one of the most used polymers in biomedical applications due to its biocompatibility, its nontoxicity and its good biodegradability. However, PEG shows a low cellular adhesion, which makes it, in principle, not suitable for cell culturing [43]. On the other, fibrin gels are composed of a mixture of two blood coagulation components, fibrinogen and thrombin [23,44]. Fibrinogen is a blood plasma protein involved in blood clotting during the coagulation process. Thrombin acts as a serine protease and initiates the polymerization reaction in which the soluble fibrinogen is converted into insoluble strands of fibrin that polymerize into a 3D organized clot [45]. The main drawback of fibrin gels is that they are extremely susceptible to protease-mediated degradation, so they cannot be used for long-term cell culturing. Thus, to overcome the limitations presented by these two materials separately, an innovative strategy consists of combining them together through a reaction named *PEGylation*. This reaction involves the terminal hydroxyl group of the PEG molecules and the amino groups of the fibrinogen protein to generate a molecular crosslinking that enhances the stability of the bonds and reduces the degradation of the material [46,47]. However, the PEGylation of fibrin hydrogels reduces cell attachment as PEG molecules chemically modify fibrin binding sides [44,47]. Despite this fact, PEGylated fibrin has been successfully used in cellular cultures [39], showing that the reduction in binding sites does not interfere with the viability of the hydrogel. Thus, the resulting semisynthetic hydrogel demonstrates an overall excellent biocompatibility, adequate cellular adhesion and sufficient stability with time to support extended cell culturing.

Despite the growing importance of PEGylated fibrin hydrogels, little is known about their mechanical properties, and even less about their evolution with time in realistic experimental conditions. Indeed, the mechanical properties of the matrix can affect axon stability and development, which can result in different neuronal networks depending on the final stiffness of the material [22,48,49]. The relative composition of thrombin and fibrinogen in the fibrin hydrogel significantly influences its bulk properties [32]. Therefore, it becomes imperative to characterize the precise mechanical properties of the resulting hydrogels and compare them to the mechanical behavior of the human brain. This evaluation is vital in determining whether the hydrogel adequately mimics the mechanical environment required for optimal neuronal growth and functionality.

Indeed, neurons in their natural in vivo environment experience mechanical forces from the brain ECM as well as interactions with neighboring cells. These mechanical cues play crucial roles in various neuronal processes, including migration, the regulation of synapse formation and the plasticity of the existing synapses. These effects are achieved through mechanotransduction, where neurons sense and respond to the biomechanical properties of their surrounding environment [24]. Considering the significant impact of mechanical forces on neuronal development and functionality, the material chosen to construct the scaffold should meet specific criteria to facilitate the healthy growth of the neuronal culture. In our study, we investigated the behavior of PEGylated fibrin scaffolds and showed that they exhibited the properties of elastic gels. These hydrogel matrices provide the necessary stiffness for neurons to attach and proliferate effectively. In turn, they create a suitable porous and hydrated environment that facilitates the dynamic flow of nutrients and waste products.

Several studies have analyzed human brain tissue to understand its viscoelastic behavior and to establish a relationship between its mechanical properties and neuronal development. Budday et al. combined compression and tension shear stress to conclude that the human brain is a nonlinear elastic tissue that dissipates stress over time and presents a compression–tension asymmetry [50,51]. Related to this, recent studies have shown that the brain Young’s modulus $E^{\mathrm{Brain}}$ depends on the analyzed region [52] and the stage of development [53], with values ranging from $E_{\mathrm{early}}^{\mathrm{Brain}}≃110$ Pa after birth to $E_{\mathrm{mature}}^{\mathrm{Brain}}≃1$ kPa in adulthood, an increase of a factor of 10 that can be ascribed to the formation and refinement of the connections within the ECM matrix. These values fall within the range of our measurements, with $E_{\mathrm{neurons},\;\mathrm{early}}^{\mathrm{PEG}-\mathrm{Fib}}≃590$ Pa and $E_{\mathrm{neurons},\;\mathrm{mature}}^{\mathrm{PEG}-\mathrm{Fib}}≃340$ Pa (Figure 5), although we observed the opposite trend, with the Young’s modulus decreasing with development. Since in our experiments we had measurements without neurons as a reference, with $E_{\mathrm{no}\;\mathrm{cells}}^{\mathrm{PEG}-\mathrm{Fib}}≃420$ Pa, we hypothesize the following scenario: Firstly, the presence of neurons makes the hydrogel stiffer, possibly due to the attachment of cells to the microscopic fabric of the scaffold in combination with the swift development of connections (axons and dendrites), effectively increasing the hydrogel Young’s modulus to about 590 Pa. We note that network formation in early developmental stages is highly dependent on the neuronal spatial arrangement and density, as observed in 2D cultures [54], which may explain the strong variability in the DIV 1–3 stage of Figure 5. And, secondly, as neurons within the hydrogel start to exhibit strong collective activity, by DIV 4–8 [54,55,56], they undergo a readjustment of connectivity that leads to the pruning of axons, which translates into a weakening of the scaffold’s cohesion and a reduction in its Young’s modulus down to about 340 Pa.

Our hypothesis is consistent with the development of neurons in their natural, brain-like environment. Spedden et al., for instance, observed that the stabilization of neurons’ microtubule cytoskeleton affects the stiffness of the brain [57]. Additionally, during the maturation of the nervous system, the mechanism known as pruning has been extensively reported, in which structural connections between neurons are removed by retracting previously formed axons. Pruning allows the neuronal network to re-evaluate its own efficiency and to selectively eliminate connections between neurons that do not exhibit a strong functional association [58]. Pruning plays a crucial role in shaping the neural circuitry, refining connectivity and optimizing the overall functionality of the nervous system as it develops. In the aforementioned study by Spedden et al. [57], they applied fluorescence and atomic force microscopy to analyze the elasticity maps of living neurons and observed that an initial increase in neuronal stiffness was followed by a subsequent decrease as a result of pruning events. This decrease in stiffness was related to the depolymerization of the microtubule structures in the connections that were removed [57]. Overall, these observations suggest a direct correlation between the mechanical properties of neurons and the structural modifications that occur during the development of neuronal networks. Thus, for our hydrogels with neurons, we attribute the sharp increase in the Young’s modulus and subsequent decrease to the intrinsic formation–pruning processes in network connectivity that are fundamental to shape a healthy and richly active neuronal culture.

PEGylated fibrin hydrogels are extremely easy to prepare and manipulate, but they are highly sensitive to the details of the preparation process. We indeed observed important differences in the rheological behavior of the hydrogel batches that slightly differed in their preparation protocols, most notably their temperature and neuronal density. For this reason, the standard error was remarkably high at some developmental timepoints (see, e.g., the strain sweep tests at DIV 1 in Figure 4). Due to this variability across repetitions, some extracted values of $G^{\prime }$ and $G^{\prime \prime }$ from the strain sweep test were considered unreliable and therefore discarded. It is noticeable that most of the variability occurred at DIV 1, which indicates that the neuronal density, and possibly the neuronal distribution across the 3D matrix, strongly dictated the initial spread of the neural connections and thus the elastic behavior of the material.

As a final remark, we note that our experiments were carried out with mouse primary neuronal cultures, but they could be extended to human induced pluripotent stem cells (hiPSCs) given their relevance in medical applications, particularly in the context of neurological disorders. However, hiPSCs require longer protocols as compared to primary cultures [56] and are more difficult to maintain in vitro. We preferred to use primary cultures to be sure that a healthy neuronal network was formed and that it remained functional for the three-week period of analysis. Nevertheless, there are already studies that explored the behavior of hiPSCs in 3D environments, such as the work of Samanta et al. [59] who considered induced cortical neurons in hyaluronic acid-based scaffolds, or the work of Adil et al., who built hiPSC-derived dopaminergic neurons in a Mebiol thermoresponsive hydrogel [60].

## 3. Conclusions

We introduced PEGylated fibrin hydrogels as competitive candidates for culturing primary neurons in a 3D matrix and rheologically characterized them in order to prove their viability to host living cells for as long as a three–week period. We observed that the 3D matrix itself, without neurons, exhibited an elastic response with a Young’s modulus $E_{\mathrm{no}\;\mathrm{cells}}^{\mathrm{PEG}-\mathrm{Fib}}≃420$ Pa that remained remarkably stable throughout the investigated time frame. Such a stability indicates the suitability of PEGylated fibrin hydrogels by themselves for long-term biomedical applications. The observed elastic behavior of the bulk material persisted when the neurons were grown inside the hydrogel, but the Young’s modulus boosted at early stages to $E_{\mathrm{neurons}}^{\mathrm{PEG}-\mathrm{Fib}}≃590$ Pa and then gradually decayed with time up to about 340 Pa. Based on the intrinsic processes related to the formation of a neuronal network, most notably the fast growth of the axons followed by intensive pruning, we conclude that the decay in the Young’s modulus is associated with natural processes within the network that are difficult to control or act on. Thus, our results show that PEGylated fibrin hydrogels are by themselves excellent materials, but one has to be prudent when growing neurons within them since the intrinsic functional development of the embedded neuronal network may alter the mechanical properties of the material with time. This aspect may be important when deciding timepoints of interest to monitor activity in such networks or when designing tailored 3D in vitro models for medical applications.

## 4. Materials and Methods

### 4.1. Hydrogels Preparation

The preparation of the primary neuronal cultures was carried out in accordance with the regulations of the Ethical Committee for Animal Experimentation of the University of Barcelona (approved ethical order DMAH-5461) and the laws for animal experimentation of the Generalitat de Catalunya (Catalonia, Spain).

Dissections were performed in a cold L-15 medium (Gibco, ThermoFisher Scientific, Waltham, MA, USA) that was previously enriched with 4% glucose 1 M, 1% glutamax (Sigma-Aldrich, St. Louis, MO, USA) and 0.4% gentamicin (Sigma-Aldrich, St. Louis, MO, USA).

Cortical tissue from CD1 mice embryos was extracted and transferred to a ‘plaiting culture medium’ (90% Eagle’s MEM enriched with 0.6% glucose, 1% 100X glutamax (Gibco, ThermoFisher Scientific, Waltham, MA, USA) and 20μg/mL gentamicin, with 5% horse serum, 5% fetal calf serum and 1μL/mL B27). Mechanical dissociation by repeated pipetting was next applied to obtain single neurons, and the cell suspension was then diluted to reach a nominal density of $10^{6}$ cells/mL.

The hydrogels were prepared by adapting the protocol from Zhang et al. [39] for their use with embryonic cortical neurons. A PEGylated fibrinogen solution was prepared by mixing 121μL of a 6 mg/mL PEG–NHS solution (713783 Sigma) with 1 mL of a 25 mg/mL fibrinogen solution (F8630-1G Sigma-Aldrich, St. Louis, MO, USA). The resulting mixture was incubated at 37 °C for 2 h to promote crosslinking between the fibrinogen and PEG strains. During the incubation process, the PEG monomers polymerized through a process known as radical polymerization, in which the characteristic polymer chain length is determined by the number of free monomers and the time elapsed for polymerization to occur. Although this process can be governed externally, the control of the final length of the PEG chains was not considered in the present study. A Thrombin solution (T4648-IKU Sigma-Aldrich, St. Louis, MO, USA) with a base concentration of 5 U/mL was then combined with the cell suspension and the PEGylated fibrinogen to prepare the final material with the following proportions: 50μL of the ‘plating medium’ with or without cells ($2\times 10^{6}$ cell/mL), 25μL of PEGylated fibrinogen and 25μL of thrombin. The role of thrombin is to cleave specific bonds in fibrinogen, resulting in the production of fibrin monomers. These monomers then polymerize through covalent bonds, forming a three-dimensional network. This whole process occurs in just 1 min, so the mixture has to be carried out all at once to ensure the homogeneous distribution of neurons within the hydrogel.

The resulting material was 100μL in volume with a final concentration of 6.5 mg/mL fibrinogen and 0.25 U/mL thrombin. A sketch of the protocol is provided in Figure 1a. In order to prepare samples that fit in the geometry of the rheometer, the 100μL hydrogel was placed in a mold made of Polydimethylsiloxane (PDMS; Dow, Corning, Midland, MI, USA) and shaped as an 8 mm diameter hollow cylinder. As described in detail in [55], at day in vitro (DIV) 5, the ‘plating medium’ was replaced with the ‘changing medium’ to limit the glial growth, and thereafter at DIV 7 to the ‘final medium’. From here onward, the ‘final medium’ was refreshed every two days. Cultures were incubated at 37 °C, 5% CO${}_{2}$ and 95% humidity. The hydrogels with and without cells were treated identically, i.e., they experienced the same manipulations and media changes.

Identical cultures as those used for rheological characterization were produced in parallel, but we incorporated the genetically encoded calcium indicator GCaMP6s during the preparation of the hydrogels. The indicator was transduced into the neurons through adeno-associated viruses, which are sufficiently small enough to diffuse across the volume of the hydrogel and access most of the neurons. This indicator becomes fluorescent in the presence of calcium, an ion involved in synaptic communication, and therefore allows for the imaging of neuronal activity. Thus, the spontaneous activity, a characteristic feature of healthy neurons in a culture, was monitored by using a Zeiss Axiovert C25 inverted microscope equipped with wide-field fluorescence microscopy. Spontaneous activity was observed from DIV 7 (when the cells started to express the calcium marker) to DIV 20, a time span that is equivalent to the one considered for rheological characterization, evincing that the cells were healthy and alive within the hydrogel matrix.

### 4.2. Hydrogels Rheological Characterization

Small Amplitude Oscillatory Shearing (SAOS) rheology was applied in order to characterize the viscoelastic properties of the hydrogel scaffolds (Figure 1b). Thus, small strain amplitudes, $\gamma_{0}\ll 1$ (100%), were used to measure the stresses associated with minor perturbations in the hydrogel structure.

Here, the material was tested under a controlled shear deformation that oscillates sinusoidally as $\gamma \left(t\right)=\gamma_{0}\sin\left(\omega t\right)$, where ω is the frequency of the oscillation. The general response of a viscoelastic material contains both in-phase and out-of-phase components, reflecting the elastic and viscous contributions, respectively. The stress response can be expressed as $\sigma \left(t\right)=\gamma_{0}\left[G^{\prime }\left(\omega \right)\sin\left(\omega t\right)+G^{\prime \prime }\left(\omega \right)\cos\left(\omega t\right)\right]$, where the term $G^{\prime }\left(\omega \right)$ is the *storage modulus* and represents the storage of elastic energy (solid-like behavior), and $G^{\prime \prime }\left(\omega \right)$ is the *loss modulus* and represents the viscous dissipation of energy (liquid-like behavior). The regime of small amplitude straining, in which the stress can be described in this form, is called the *linear viscoelastic regime* (LVR) [37].

#### 4.2.1. Determination of Rheological Properties: Data Analysis

The measured $G^{\prime }$ and $G^{\prime \prime }$ modulus allowed for the definition of the *complex shear modulus*, $G^{*}=G^{\prime }+iG^{\prime \prime }$, that accounts for the entire viscoelastic response of the material. Being a complex number, it is possible to extract its modulus $|G^{*}|$ and its phase δ since $G^{*}=\left|G^{*}\right|e^{i\delta }$. These two quantities provided useful insight in the material behavior.

The *complex modulus*, expressed as:

(1) $$\begin{array}{c} |G^{*}|=\sqrt{{\left(G^{\prime }\right)}^{2}+{\left(G^{\prime \prime }\right)}^{2}}\;, \end{array}$$

gives information about the shear stiffness of the material [61]. Interestingly, $|G^{*}|$ can be related to the the Young’s modulus, *E*, which quantifies the relationship between the tensile (or compressive) stress and the axial strain in the linear elastic region. Knowing $|G^{*}|$, *E* can be obtained as:

(2) $$\begin{array}{c} E=2|G^{*}|\left(1+\nu \right)\;, \end{array}$$

where ν is the Poisson’s ratio [37,62]. For the fibrin matrices, the Poisson’s ratio was taken as ν≃0.25 [63].

On the other hand, the *phase shift* δ, defined as: [37,64]

(3) $$\tan\delta =\frac{G^{\prime \prime }}{G^{\prime }}\;,$$

provides information about the tendency of the material to behave as elastic or viscous. For δ=0 rad, the material response is the one of an ideally elastic material, while for δ=π/2≃1.57 rad, the material response is one of a perfect viscous liquid. Intermediate phase shifts reflect different degrees of viscoelastic behavior.

#### 4.2.2. Rheological Tests

Three different tests were performed on the PEGylated fibrin hydrogels.

1. **Time sweep**: This rheological test was used to track the evolution of the hydrogel structure along time and procure information such as the degradation, gelation or solvent evaporation. The oscillation frequency, ω, and the strain amplitude, γ, were kept constant in this test. In the present study, they took values of 2π rad/s (1 Hz) and 5%, respectively (see Table 1).
2. **Strain sweep**: The amplitude of the strain oscillation, γ, is changed periodically while the frequency, $\omega_{0}$, remains constant (see Figure 1b). This test is performed to obtain information about the linear viscoelastic region (LVR). As a consequence of the linear response and the small deformations, the test can be carried out without damaging the microscopic structure of the sample, which is crucial to keep the scaffold and the inner neural network intact. For these experiments, ω was fixed to $\omega_{0}=2\pi$ rad/s (1 Hz) and γ was progressively increased from a 0.1 to 100% strain.
3. **Frequency sweep**: The oscillation frequency, ω, is progressively increased at a constant strain amplitude $\gamma_{0}$ (see Figure 1b). This test provides information about the rheological response of the hydrogel at different timescales and reveals whether the sample softens or thickens at faster deformations. The tests are performed at a selected $\gamma_{0}$, ensuring that the sample remains in the LVR. For the present work, $\gamma_{0}$ was fixed at 5%, and ω increased from 0.1 to 100 rad/s.

A bulk rheometer HR-2 Discovery (TA instrument) was used with an 8 mm plate–plate geometry and a fixed gap of 500μm. The gap space for these rheological tests was chosen to ensure the integrity of the samples. Preliminary tests were carried out in which the geometry of the rheometer was vertically displaced to squeeze the sample until the normal stress reached a value of ≃0.2 N. Sandpaper (grit P600-BF08, Wurth, Künzelsau, Germany) was attached to both surfaces of the plate–plate geometry to prevent the hydrogel from slipping during the measurements [65]. A handmade solvent hood was used to avoid the evaporation of the liquid contained in the hydrogel during the test. All tests were performed at a constant temperature of 37 °C, the physiological temperature of living neurons. The hydrogels were tested at different days in vitro with and without neurons. Each sample was used only for one test, and each test was repeated at least three times for statistical purposes.

### 4.3. Immunochemistry

The PEGylated fibrin hydrogels cultured with neurons were fixed with a 5% Paraformaldehyde solution (F8630-1G Sigma-Aldrich, St. Louis, MO, USA) in PBS for 30 min at room temperature, rinsed twice with PBS and incubated with a blocking solution containing 2% Triton (F8630-1G Sigma-Aldrich, St. Louis, MO, USA) and 5% Normal Goat Serum (Gibco, ThermoFisher Scientific, Waltham, MA, USA) in PBS for 2 h at room temperature and placed in a shaker (battery–powered Shaker KM 2, Carl Roth, Karlsruhe, Germany) at 75 RPM. A primary antibody against actinin (F8630-1G Sigma-Aldrich, St. Louis, MO, USA) was applied diluted in a blocking solution (1:300) and incubated overnight at 4 °C while shaking. After rinsing 6 times with PBS, the Alexa568-conjugated secondary antibody against mice (Gibco, ThermoFisher Scientific, Waltham, MA, USA) was diluted in a blocking solution (1:400) and incubated for 2 h at room temperature. The 3D cultures were then rinsed with PBS and mounted by using DAPI-fluoromount–G (1:500) (ShouternBiotech, Birmingham, AL, USA) and a CellMask Red Plasma Membrane Stain (Gibco, ThermoFisher Scientific, Waltham, MA, USA) for 1 h at 75 RPM at room temperature. Immunocytochemical images were acquired on a confocal microscope (LSM800, Zeiss, Oberkochen, Germany; MicroFabSpace and Microscopy Characterization Facility, Unit 7 of ICTS “NANBIOSIS” from CIBER-BBN at IBEC).

## Figures and Tables

**Table 1:** Summary of the parameter values used to perform the three different oscillation tests. Hydrogels with and without cells are measured at different DIV along three weeks. All test were performed at 37 °C with a 500μm gap.

Parameter	Time Sweep	Strain Test	Frequency Sweep
Frequency ω (rad/s)	2π	2π	0.1–100
Strain γ (%)	5	0.1–100	5

## Data Availability

Data are available upon request to the authors.

## Abbreviations

The following abbreviations are used in this manuscript:

2D	Two dimensional
3D	Three dimensional
ECM	Extracellular matrix
AAV	Adeno-associated virus
DIV	Day in vitro
MEM	Modified Eagle Medium
E16	Embryonic day 16
PEG	Polyethylene glycol
PEG-NHS	Mono-Methyl polyethylene glycol succinate N-succinimidyl ester
PDMS	Polydimethylsiloxane
SAOS	Small Amplitude Oscillatory Shearing
LVR	Linear viscoelastic regime
$G^{\prime }$	Storage modulus
$G^{\prime \prime }$	Loss modulus
$G^{*}$	Complex shear modulus
δ	Phase shift
*E*	Young’s modulus
ν	Poisson’s ratio
ω	Oscillation frequency
γ	Strain amplitude
PBS	Phosphate-buffered saline
RPM	Revolutions per minute
